# The Link between Chronic Stress and Accelerated Aging

**DOI:** 10.3390/biomedicines8070198

**Published:** 2020-07-07

**Authors:** Yegor E. Yegorov, Anastasia V. Poznyak, Nikita G. Nikiforov, Igor A. Sobenin, Alexander N. Orekhov

**Affiliations:** 1 Engelhardt Institute of Molecular Biology, Russian Academy of Sciences, Moscow 119991, Russia; yegorov58@gmail.com; 2 Institute for Atherosclerosis Research, Skolkovo Innovative Center, Moscow 121609, Russia; 3 National Medical Research Center of Cardiology, Institute of Experimental Cardiology, Moscow 121552, Russia; nikiforov.mipt@googlemail.com (N.G.N.); igor.sobenin@gmail.com (I.A.S.); 4 Institute of Gene Biology, Center of Collective Usage, Moscow 119334, Russia; 5 Laboratory of Angiopathology, Institute of General Pathology and Pathophysiology, Moscow 125315, Russia; 6 Institute of Human Morphology, 3 Tsyurupa Street, Moscow 117418, Russia

**Keywords:** inflammaging, ROS, mitochondria, oxidative stress, mitophagy, uncoupling, antioxidants, macrophage

## Abstract

People exposed to chronic stress age rapidly. The telomeres in their cells of all types shorten faster. Inflammation is another important feature of stress that, along with aging, accounts for the phenomenon of inflammaging. In addition to aging itself, inflammaging can contribute to the development of several pathologies, including atherosclerosis, diabetes, hypertension, and others. Oxidative stress is one of the main mechanisms related to stress. Oxidative stress is caused by the over-production of reactive oxygen species (ROS) that can damage various tissues. The main source of ROS is mitochondria. Being suppressed by mitochondrial mutations, mitophagy can aggravate the situation. In this case, the aging-specific pro-inflammatory changes are amplified. It happens because of the inability of cells to maintain the normal state of mitochondria. Macrophages are the crucial element of the innate immunity associated with the chronic inflammation and, subsequently, with the inflammaging. In this review, we focus on the therapy approaches potentially reducing the deleterious effects of oxidative stress. These include stimulation of mitophagy, activation of mitochondrial uncoupling, induction of the expression of the telomerase catalytic component gene, and use of antioxidants. Any method reducing oxidative stress should improve post-traumatic stress disorder.

## 1. Psychological Stress and Aging

Psychological stress is considered to be an important risk factor for numerous diseases. The common feature of these pathologies is cellular senescence, which causes functional alterations and is associated with cancer and cardiovascular, neurodegenerative, and autoimmune disorders. All of these conditions are usually associated with whole-body aging, but, in the case of lasting severe stress, they can occur early in life [1,2].

**Figure 1:**
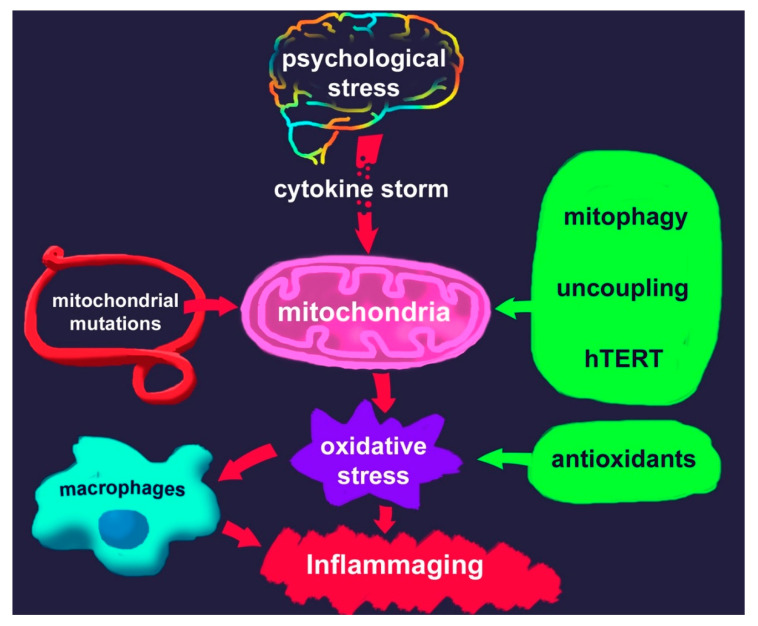
Scheme of the association between psychological stress and inflammaging through oxidative stress; potential interventions to reduce the resulting detrimental effects are listed on the right.

Numerous studies have shown a link between chronic psychological stress and mental disorders, such as major depressive disorder, and post-traumatic disorder (PTSD), as well as accelerated aging. This suggests the involvement of neural, physiologic, molecular, and genomic mechanisms. Chronic psychological stress is also believed to stimulate pro-inflammatory cytokines release (Figure 1) [3], thus triggering inflammation [4]. Accordingly, antidepressants were shown to reduce enhanced levels of cytokines [5].

The hypothesis that oxidative stress stimulates inflammation is well known and is supported by a sufficient body of evidence. Based on it, oxidative stress was also suggested to trigger a crosstalk between the immune and the central neural systems within the human organism. This influence may have a crucial impact on emotional wellbeing [6].

Reactive oxygen species (ROS) overproduction can lead to cellular damage and macromolecule metabolism alterations that, in turn, are associated with the development of aging phenotypes [7]. Further investigations focused on the missing links between oxidative stress and telomere length. Initially, research was based on in vitro studies using cultured cells that demonstrated the association between oxidative stress inductors and premature senescence [8]. Epel et al. discovered the existence of the association between oxidative stress and accelerated telomeres shortening in humans in 2004 [9]. They analyzed the impact of psychological stress on pre-menopausal women. Although this study was small, it laid the groundwork for further analyses.

In vivo studies performed so far indicate that, in Parkinson’s disease [10] and diabetes type II [11], there is a clear association between selected biomarkers of oxidative stress-induced damage and the length of the telomeres, which was not observed in healthy individuals. In addition, Starr et al. demonstrated a possible association between two aging-related single-nucleotide polymorphisms (SNPs) of oxidative stress genes and telomere length [12].

A recent study examined the association between redox-state markers and telomere length in blood and vascular tissue in coronary artery disease. Tissue-specific inverse associations between superoxide production and telomere length were identified. However, no association was found between superoxide dismutase activity and telomere length in vascular and blood cells [13].

To sum up, the existing data indicate that the association between oxidative stress and telomere shortening may be more complicated than originally conceived. Differences in research data may be explained by clinical and ethnical diversity of the subjects and by inadequate choice of the tested biomarkers.

## 2. Cell Senescence

Speaking of aging, it is worth mentioning the cellular aging and senescence. Although the meaning of the term “cell senescence” seems obvious as it was first used to indicate the complex of processes that accompanies cell proliferation arrest in culture, it appears to be more complicated. Now it is clear that cell senescence is not just the exhaustion of cells proliferative potential, but it is a lasting arrest. Among the causes are various injuries, all kinds of stresses [14], a conflict of regulation during the activation of certain oncogenes, and even phenomena accompanying the wound process [15] and normal embryonic development [16].

Previously, Leonard Hayflick reported the limited proliferative potential of human cells, which later became known as the “Hayflick limit” [17]. He also suggested that the reaching of the Hayflick limit is related to in vivo aging. Later, the telomeric theory of aging was suggested, which explains the nature of the Hayflick limit. Further research in the field of cytogerontology revealed that the under-repair of telomeres is of great importance. It was shown that telomeric DNA loss increases under conditions of oxidative stress. Thus, it appeared that the free-radical theory could be combined with the telomeric theory, and under-repair was added to under-replication [18].

Oxidative stress was demonstrated to stimulate the loss of telomeric DNA, and the processes resulting from telomeric DNA loss were shown to be similar to those occurring during cellular senescence [19]. A crucial role for mitochondria in the determination of telomere shortening rate was proposed due to their leading role in the ROS generation [20]. Moreover, a decrease in the number of mitochondria in cells was shown to lower the number of senescent cells in vivo [21].

It is essential to emphasize that there are several limitations in evaluating cell senescence by measuring telomere length. First, cell senescence can be local or generalized. By measuring the length of telomeres in blood cells, it is impossible to judge small local processes that occur in any specific pathologies, including, for example, pathologies limited to any region of the brain.

Second, changes in telomere length in blood cells can be transient [22]. The reason for this is unclear, but two possibilities can be assumed, i.e., the increase in telomerase activity that lengthens telomeres, or the exit from the acute stage of stress. The fact is that the maturation of hematopoietic stem cells to blood cells is long (when measured by the number of cell divisions). In conditions of acute need, the body triggers the proliferation of progenitor cells, not of stem cells. If this stimulus and resulting proliferation occur under stress conditions, then there is a noticeable shortening of the telomeres. At the end of stress, slowly, due to the proliferation of stem cells that were not affected (they very rarely divide and are located in a special hypoxic niche), telomere length can be restored.

The third limitation is that most measurements of telomere length are made via PCR. This is the easiest and cheapest way. At the same time, all the information collected concerns the average size of telomeres. However, cell senescence is determined by the shortest telomeres, which signal about DNA damage, leading to a stop in cell proliferation and initiating senescence. When analyzing telomere length using PCR, it is assumed by default that telomere size variance is the same in control and experimental samples.

## 3. Mitochondria and ROS Production

As mentioned above, oxidative stress is an important stimuli of the telomere shortening, which makes ROS and ROS-related enzymes a valuable part of the process of cellular senescence. ROS and reactive nitrogen species (RNS) normally participate in numerous redox reactions within the cell. However, the overproduction of ROS and RNS is associated with oxidative stress that can cause cellular damage, development of inflammation, and severe disorders. ROS also causes an irreversible progression of oxidative decay, promoting the impairment of physiological functions, increasing disease incidence, and reducing the life span [23].

Oxidative stress can be caused not only by the excessive production of ROS but also by insufficient activity of endogenous systems fighting against the oxidative attack [24,25].

**Figure 2:**
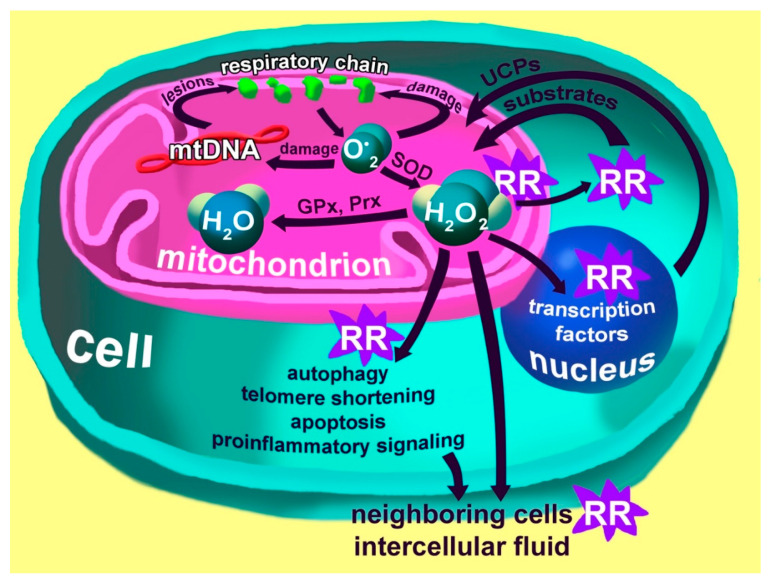
Generation of reactive oxygen species (ROS) by the mitochondrial respiratory chain. Superoxide anions generated by the respiratory chain damage the respiratory chain itself and the mitochondrial DNA, which leads to changes in the respiratory chain that increase the production of the superoxide anion. Superoxide anions also damage the mitochondrial membranes, causing lipid peroxidation and leading to changes in membrane properties. Special superoxide dismutase (SOD) enzymes convert superoxide anions into hydrogen peroxide, which is then converted to water by peroxiredoxins (Prx) and glutathione peroxidase (GPx). Hydrogen peroxide plays a major redox-regulating (RR) role in this system. In mitochondria, it changes the activity of enzymes, in the cytosol, it affects the delivery of substrates into the mitochondria, whereas, in the nucleus, it alters the activity of transcription factors, causing the response kernel, which is reflected in increased protein expression of uncouplers (UCPs). UCPs are transported to the mitochondria, where they reduce the proton potential of the inner membrane, thereby reducing the production of the superoxide anion. Excessive production of hydrogen peroxide in the cytoplasm triggers autophagy processes, causes shortening of telomeres, potentially starts the process of apoptosis and causes increased expression of pro-inflammatory factors that can affect neighboring cells and the whole organism [27].

Normally, the greatest source of ROS production is the mitochondria as a result of the activity of the respiratory chain and oxidoreductases (Figure 2). ROS can also be generated by peroxisomes, endoplasmic reticulum, plasma membrane, cytosol, lysosomes, microsomes, and nuclear envelope [26].

Several circumstances are crucial for ROS generation in mitochondria. One of the most important factors is the potential of mitochondria inner membrane, the decrease of which results in a reduced generation of ROS. The two most obvious mechanisms of this process are a local decrease in oxygen concentration and a slowdown of electron transport. Uncoupling increases respiration, which reduces the local concentration of oxygen and therefore the production of ROS [28,29].

A close connection between the development of psychological stress and ROS production is underlined by changes in the activity of enzymes involved in ROS inactivation. ROS metabolism involves various genes, among which are glutathione S-transferase mu 1 and 2 (GSTM1 and GSTM2). Their transcripts are modified according to PTSD progression and are also contributed to a risk of PTSD development [30]. In addition, it has been shown that the expression level of thioredoxin reductase (TXNRD1) may be enhanced in PTSD patients compared to control individuals exposed to trauma [31].

The production of ROS by mitochondria depends on the activity of the uncoupling proteins. Various genetically programmed variants of these proteins are associated, on the one hand, with the intensity of oxidative stress and shortening of telomeres [32] and, on the other hand, with the overall life expectancy.

## 4. The Special Role of Mitochondria and Macrophages in the Development of Chronic Inflammation

As depicted in Figure 2, mitochondria are the main producers of ROS, but, at the same time, they can also be harmed by an increased ROS concentration. There are a few possible scenarios. A defect in mitochondria is another potential consequence of ROS influence. It inhibits the utilization of the defective mitochondria preventing mitophagy or the mitochondrial fission process, which often precedes mitophagy. These mitochondria become a permanent source of ROS over time, and the cell undergoes accelerated aging and acquires senescence-associated secretory phenotype (SASP). At some point, the mitochondria are destroyed. In case they cannot be utilized properly (mitophagy), the innate immune system, in concert with inflammatory components, recognizes the mitochondrial DNA as an object of attack. It is believed that mitochondria can stimulate the innate immune system because of their bacterial origin. If such events occur with the macrophage, it becomes a permanent source of pro-inflammatory factors; the process increases over time and cannot be stopped. Such macrophage reactions can contribute to the development of atherosclerosis, osteoporosis, and neurodegenerative processes. Genetic analysis of mitochondrial DNA, in addition to conventional genetic analysis, can be useful for patients suffering from stress disorders to identify those most vulnerable to oxidative stress.

## 5. Oxidative Stress and the Pro-Inflammatory Phenotype of Immune Cells

The immune system is not an exception regarding cell senescence. As supported by the majority of experimental data, immunosenescence involves the deterioration of the innate and adaptive immune response efficacy, although the elements of the immune system are altered with aging in different ways.

Several features accompany the senescence of immune cells, among which are the termination of proliferation, telomeres shortening, morphological changes, activity of the SA-βGal enzyme, and also SASP. SASP is associated with the ability to secrete pro-inflammatory cytokines [33].

### 5.1. Innate Immunity

Many age-related alterations in neutrophil function have been described. Functions, such as the production of free radicals, intracellular killing, apoptosis, and chemotaxis, decline. In addition, the release of pro-inflammatory cytokines is enhanced, and this may promote inflammaging. Notably, adhesion and phagocytosis appear not to change with aging [34].

Decreased level of major histocompatibility complex (MHC) class II human leukocyte antigen DR-isotype (HLA-DR) molecules and decreased activation ability of macrophages were observed as a result of aging [35]. The generation of reactive nitrogen and oxygen intermediate products, phagocytosis ability, and respiratory burst also decrease, which indicates the weakening of the microbicidal activity of macrophages [36]. In addition, the minor population of CD14^+^CD16^+^ monocytes were observed to be significantly enhanced in elderly subjects. Although the number of membrane receptors responsible for cell activation does not decrease during aging, signaling pathways are affected [37]. These include phosphoinositide 3-kinases (PI3K), mitogen-activated protein kinase (MAPK), the Janus kinase-signal transducer and activator of transcription (Jak/STAT) pathways, and others [38].

The changes in Toll-like receptor (TLR) function deserve particular attention [38]. The lowering of TLR-1 expression and a subsequent decrease in the levels of CD80 and CD86 expression is accompanied by a decrease of cytokines release. Conversely, the stimulation of TLR4 in CD14^+^CD16^+^ inflammatory monocytes was shown to enhance cytokine generation, which can be explained by the persistent activity of NF-κB [37].

Aging affects the expression of surface receptors in natural killer (NK) cells. The expression of cytotoxicity-activating receptors was shown to be decreased and, consequently, the cytotoxic potential of single cells was reduced. The total number of NK cells increases with aging, and this may have a compensatory value. The proportion of different phenotypes of NK cells is also affected. Thus, the proportion of CD56^bright^ NK cells decreases, and the proportion of CD56-NK cells increases [39]. Notably, antibody-dependent cellular cytotoxicity capacity, CD16 expression, and some other parameters are not influenced by aging.

### 5.2. Adaptive Immunity

Phenotypes and functions of both T and B cells are more vulnerable to aging than those of the cellular components of innate immunity. This makes age-related detrimental changes more frequent in the adaptive immune response [40]. OCTO and NONA studies tracked persistent changes in the immune response related to aging. The aim of these studies was to cluster immune parameters, such as poor T cell proliferation, low B cell number associated with the so-called immune risk phenotype (IRP), inversion of the CD4/CD8 ratio, expansion of late differentiated CD8+ T cells, and cytomegalovirus (CMV) seropositivity [41]. Unfortunately, the link between IRP and increased mortality has not been replicated.

Decreased avidity and quantitative decrease of the antibody response were demonstrated in elderly subjects, along with decreased efficacy. This was found to be the consequence of the limited repertoire diversity of memory B cells [42].

Concerning T cells, we are facing phenomena called memory inflation, which result from naïve T cell activation following exposure to antigens and insufficient renewal of the naïve cellular pool throughout the lifespan [43].

The long-lasting antigenic stimulation causes the accumulation of memory cells according to the antigens to which the cells were previously exposed, in which effectiveness decreases with time [44]. This causes the progressive filling of the immune space and reduces memory cell functions, such as clonal expansion and IL-2 production. Alternatively, it increases interferon-gamma (IFNγ) that has cytotoxic functions and accumulates in inflammatory lesions. All these modulations are associated with changes in CD28 receptor signaling and other signaling pathways [45]. To compensate for the insufficient production of naïve T cells due to thymic involution, already existing naïve cells undergo peripheral homeostatic proliferation. T regulatory cells may also be affected both quantitatively and qualitatively, which causes important alterations in the host immune response [46]. PTSD is an example of the disease that involves oxidative stress. Oxidative stress affects immune cells, favoring their pro-inflammatory phenotype. PTSD is also characterized by a phenomenon called oxi-inflamm-aging or inflammaging [47].

## 6. Inflammaging

During the aging process, several cytokine-related effects are observed. In particular, anti-inflammatory cytokines levels decrease, while the levels of pro-inflammatory cytokines (interleukin-1 (IL-1), IL-6, tumor necrosis factor alpha (TNF-α), and others) grow. The complex of these changes in combination with alterations of the innate immune response was termed “inflammaging”. The state of inflammaging is characterized by subclinical inflammation caused by alterations in immune responses. Serum tests of aging subjects revealed only a small increase in the levels of pro-inflammatory cytokines [48].

Inflammaging is now considered the consequence of immunosenescence, which implies the inappropriate response of adaptive immunity to pathogens exposure and other types of chronic stress in aging subjects [49].

## 7. Potential Approaches to Reduce Oxidative Stress in Stress Disorders

### 7.1. Potential Anti-Inflammatory Treatment Strategies

Today, only a few drugs with the properties of selective inhibitors of serotonin reuptake (SSRIs) are approved by US Food and Drug Administration and other organizations for the therapy of stress-related disorders. Their effectiveness is limited [50].

The effectiveness of cyclooxygenase 2 inhibitors is high in animal models, but, in the case of human disorders, it remains to be determined [51].

Cytokine-blocking antibodies have not been tested. To inhibit cytokines, monoclonal antibodies are often used for the treatment of malignancy and autoimmune disorders. Many compounds of this class are approved, and several of them were shown to be effective as the therapeutic approach for the depression. Among such agents are infliximab, adalimumab (anti-TNF-α antibodies), and tocilizumab (anti-IL-6 receptor antibody).

Several studies on the use of glucocorticoids have shown some effectiveness [52].

Angiotensin-converting enzyme inhibitors have anti-inflammatory properties and were shown to reduce PTSD symptoms [53].

Beneficial effects on the stress-related disorders, such as PTSD, were described as the result of a healthy lifestyle. This includes a healthy diet and physical activity. However, these results are unclear due to the unaccounted differences in sampling [54,55]. Intriguingly, there is increasing evidence that physical activity and exercise can reduce inflammation [56].

It should be noted that inflammation is the evolutionary conserved response of the body aimed at restoring the status quo. It is a “good” reaction. However, sometimes this reaction does not achieve its goal. For example, a too intense inflammation has great destructive power and damages organs and tissues, whereas a too weak inflammation does not reach its goal and can turn into a chronic state. Direct artificial suppression of inflammatory processes, especially in the case of unclear pathophysiological mechanisms, can harm the course of a disease. In the case of PTSD, it is only known that the disease occurs due to an incorrect reaction of the body to stress.

A recent study encourages us to take a fresh look at the possible ways of PTSD development [57]. A statistically significant association of a weak inflammatory response in the acute phase of trauma with subsequent development of PTSD was found. This investigation once again highlights the fact that applying anti-inflammatory therapy in the case of PTSD can be dangerous.

Serious changes in the concentration of various cytokines occur during the development of PTSD (cytokine storm; see Figure 1). These changes generally lead to increased production of reactive oxygen species in various cell types, including cells of the immune system, vascular wall, and nervous system. The entire body is affected by oxidative stress and begins to age rapidly. If it is impossible to target inflammation, it is possible to reduce oxidative stress. Notably, anti-aging measures (regarding lifestyle, diet, exercise, etc.; see above) also have a positive effect on PTSD symptoms.

### 7.2. Activators of Mitophagy

Autophagy is directed at digesting useless components and those that work improperly in cells. The special type of autophagy aimed at the degradation of damaged or dysfunctional mitochondria is called mitophagy. Activation of mitophagy is one of the ways to reduce the oxidative stress due to mitochondria activity in oxidative stress-associated ROS production [58].

Pink1, a PTEN-induced kinase, is attracted to the mitochondrial membrane in response to the loss of membrane potential that is associated with mitochondrial damage. There, Pink1 phosphorylates and activates an ubiquitin ligase called parkin, which, in turn, recruits the autophagy adaptor protein p62. This causes the encapsulation of mitochondria in autophagosomes with the participation of LC3 [59].

Cellular ROS production was shown to increase in response to the knockout of *Pink1* and other mitophagy-related genes. This suggested that ROS are generated by mitochondria that escaped mitophagy [60]. Resveratrol, a sirtuin 1 (SIRT1) activator, was reported to stimulate mitophagy and thus to reduce oxidative stress in mdx mice [61].

Activation of sirtuin-3 (SIRT-3) was reported to have a positive impact on the stimulation of mitophagy [62]. Among SIRT3 activators, dihydromyricetin and honokiol were tested in various conditions related to mitochondrial functional impairments. Dihydromyricetin was shown to suppress chondrocyte degeneration in osteoarthritis [63]. Honokiol was reported to activate SIRT3 in a study dedicated to the treatment of intervertebral disc degeneration [64].

### 7.3. Uncouplers

Uncouplers increase the permeability to protons of the inner mitochondrial membrane, which leads to a decrease in the proton gradient. The significant decrease in the proton gradient complicates ATP generation mediated by complex V, located in the inner membrane of mitochondria, which uses the proton gradient. Thus, energy is consumed as heat, which results in the separation of oxidation and phosphorylation.

Uncouplers were shown to suppress the generation of ROS [65]. An increase in uncouplers production was reported to be stimulated by ROS generation. ROS production, in turn, can be lowered by decreasing the proton gradient in response to uncouplers [66].

The function of UCP1, the thermogenin protein from brown fat, is quite well established. Thermogenesis reduces cold-induced ROS production in the presence of oxidative modification of UCP1 at the cysteine residue 253. Thermogenesis can be blocked by the mitochondrial antioxidant MitoQ or by the restoration of the redox potential with N-acetylcysteine [67].

Dinitrophenol (DNP) and niclosamide are uncouplers that work as weak acids, providing an H^+^ source for the lowering of the mitochondrial membrane potential [68]. Interestingly, DNP has been known for more than 120 years and prescribed as an anti-obesity drug [69].

DNP has been shown to increase the lifespan of mice. At the same time, an increase in tissue respiration, weight loss, and a decrease in plasma glucose and triglycerides were observed [70]. DNP also caused an increase in the average lifespan of flies, without affecting the maximum value [71].

### 7.4. hTERT Activators

Since the stress is accompanied by accelerated aging, telomerase induction should reduce the effects of aging by lengthening the telomeres.

Telomerase has numerous functions within the cell besides telomere stabilization, but the function of the catalytic subunit hTERT in mitochondria is not clear yet. However, recent studies provide evidence of the participation of hTERT in the reduction of oxidative stress-induced damage [72]. Reduced telomerase activity can be restored by various mechanisms. PI3K/Akt, MAPK/ERK1/2, and the Wnt/β-catenin pathways were shown to upregulate hTERT expression and/or activity [73].

Cycloastragenol, a telomerase activator, was reported to transiently activate telomerase in T-lymphocytes, neonatal keratinocytes, and fibroblasts [74]. This substance received the commercial name TA-65 and has been sold as a food supplement since 2013. It works through the activation of the ERK pathway, enhancing the expression of telomerase. Telomeres lengthening induced by cycloastragenol was not accompanied by an increase in cancer incidence [75]. However, not many studies have evaluated the positive impact of this drug, and more detailed investigations are needed.

Resveratrol was shown to activate telomerase in addition to mitophagy. It also seems to act through the activation of SIRT1 [76]. Despite the well-known antioxidant properties, resveratrol can have also prooxidant effect. This was proven in various researches that revealed different properties of resveratrol depending on the cell type and concentration [77,78]. Moreover, resveratrol was demonstrated to have apoptosis-inducing properties in the melanoma cell line, A375SM. It is implemented through the stimulation of ROS generation [79]. The co-transfection of bone marrow mesenchymal stem cells of rats with telomerase and nerve growth factor displayed a positive impact on learning and memory [80].

### 7.5. Antioxidants

The use of mitochondria-targeted antioxidants is probably the most obvious approach to reduce ROS production. It turned out that these antioxidants, for example, SkQ1, can marginally increase the lifespan of mice and lower the severity of many aging-associated pathologies, although MitoQ was demonstrated to increase the proliferative potential of cells only in the presence of minor oxidative stress [81].

Unfortunately, numerous studies revealed no effect of antioxidants on cell proliferation in culture. This makes the understanding of the underlying processes controversial. However, the recent work of Liao et al. demonstrated the inhibition of cell senescence in adipose tissue-derived mesenchymal stem cells (ADSCs) by reduced glutathione and melatonin [82].

These contradictions reflect the limitations of the free-radical theory of aging. Cell culture is a kind of pure isolated system that does not provide all stress stimuli normally present within a living organism. This is proven by the significant enhancement of the lifespan of mice who were held in normal vivarium conditions, in contrast to what observed from mice kept in a pathogen-free vivarium, in which antioxidants showed no effect [83].

The most beneficially seems to be the diet with natural antioxidants, which have the potential to reduce the oxidative stress and thus to decrease an inflammation rate.

## 8. Conclusions

The ideal treatment for stress-related disorders has to be based on the knowledge of the mechanisms causing each disorder operating at the nervous system level. It should aim at “erasing” the memory of an event, or at least at blocking newly formed, easily aroused connections between brain structures. Research in this direction is underway, but it is still far from practical implementation.

Under these conditions, it is possible to try blocking the negative consequences of these diseases development. The main pathogenetic factor that can be involved in is oxidative stress, which is associated with inflammation and occurs as a result of psychological stress.

The stress response is an adaptive mechanism that allows mobilizing all the resources of the body to fight a temporary threat. When the process becomes chronic, the stress associated with adaptive mechanisms becomes destructive. Glucocorticoid receptors are present in most cells of the body, so the stress response is very generalized. Cells receive a huge number of signals that significantly affect their physiology. In the case of immune system cells, this process is even more pronounced. Cellular responses to such essential signals are almost always associated with redox regulation, which involves mitochondria as generators of the oxidative reaction components. As a result, the entire body falls under conditions of oxidative stress. Conditions are created for accelerated aging and the development of a wide variety of pathologies associated with aging. During the development of such pathologies, senescent cells arise, which, in turn, acquire SASP and begin to secrete pro-inflammatory components into the surrounding space. Thus, the overwhelming effect on oxidative stress can break this vicious circle and reduce the deleterious effects of psychological stress.

In principle, two strategies are possible to control oxidative stress: the inactivation of already formed ROS and RNS or the reduction of their formation. The second option is preferable. In this view, antioxidants are less preferable than therapies reducing ROS production.

Reducing ROS production can be achieved by removing dysfunctional mitochondria, which become autonomous sources of ROS, promoting damage to cells and their environment. This can be achieved by enhancing mitophagy, i.e., by delivering such dysfunctional mitochondria to lysosomes for complete disposal. Unfortunately, defective mitochondria that produce excess ROS can maintain a high membrane potential for a long time and thus be inaccessible to the natural mechanism of mitophagy targeted at removing mitochondria with very low membrane potential.

Only in the 21st century has it become clear that, in response to oxidative stress, the protein component of telomerase (hTERT) migrates from the cell nucleus to the mitochondria and increases cell survival. The mechanism of this process is still unclear, but it is very tempting to induce hTERT expression in cells to resist oxidative stress. Unfortunately, natural selection has very significantly suppressed hTERT expression in human cells, and 30 years of attempts to stimulate hTERT expression have yielded only minor results. From a formal point of view, hTERT is an oncogene that can cause tumor growth of already altered, precancerous cells.

The molecules most suitable for practical use are uncouplers. Their effectiveness against oxidative stress is well proven; however, it is necessary to develop compounds with reduced and prolonged tissue-specific activity. Possible candidates may be free fatty acids, which are natural uncouplers. In addition to reducing oxidative stress, uncouplers can reduce weight and lower the plasma concentration of glucose and triglycerides, thereby reducing the likelihood of developing metabolic syndrome, type 2 diabetes, and atherosclerosis, which are diseases of our modern civilization.
